# Advances in Key Drug Target Identification and New Drug Development for Tuberculosis

**DOI:** 10.1155/2022/5099312

**Published:** 2022-02-25

**Authors:** Jie Mi, Wenping Gong, Xueqiong Wu

**Affiliations:** Tuberculosis Prevention and Control Key Laboratory/Beijing Key Laboratory of New Techniques of Tuberculosis Diagnosis and Treatment, Senior Department of Tuberculosis, The 8th Medical Center of PLA General Hospital, Beijing 100091, China

## Abstract

Tuberculosis (TB) is a severe infectious disease worldwide. The increasing emergence of drug-resistant *Mycobacterium tuberculosis* (*Mtb*) has markedly hampered TB control. Therefore, there is an urgent need to develop new anti-TB drugs to treat drug-resistant TB and shorten the standard therapy. The discovery of targets of drug action will lay a theoretical foundation for new drug development. With the development of molecular biology and the success of *Mtb* genome sequencing, great progress has been made in the discovery of new targets and their relevant inhibitors. In this review, we summarized 45 important drug targets and 15 new drugs that are currently being tested in clinical stages and several prospective molecules that are still at the level of preclinical studies. A comprehensive understanding of the drug targets of *Mtb* can provide extensive insights into the development of safer and more efficient drugs and may contribute new ideas for TB control and treatment.

## 1. Introduction

Tuberculosis (TB) is a respiratory infectious disease caused by *Mycobacterium tuberculosis* (*Mtb*). The World Health Organization (WHO) reported approximately 9.9 million incident cases and 1.28 million deaths related to TB in 2020 [1]. Although a declining trend in the incidence and mortality of TB has been observed since 2010, the global TB burden remains a challenge. In addition, multidrug-resistant (MDR)-TB poses a threat to TB control. For more than 10 years, approximately 3-4% of new TB cases and 18-21% of patients with TB with retreatment had MDR-TB or rifampicin (RFP)-resistant TB (RR-TB) [1]. Therefore, the importance of preventing *Mtb* transmission and identifying treatments for MDR-TB and XDR-TB must be recognized and addressed.

**Figure 1:**
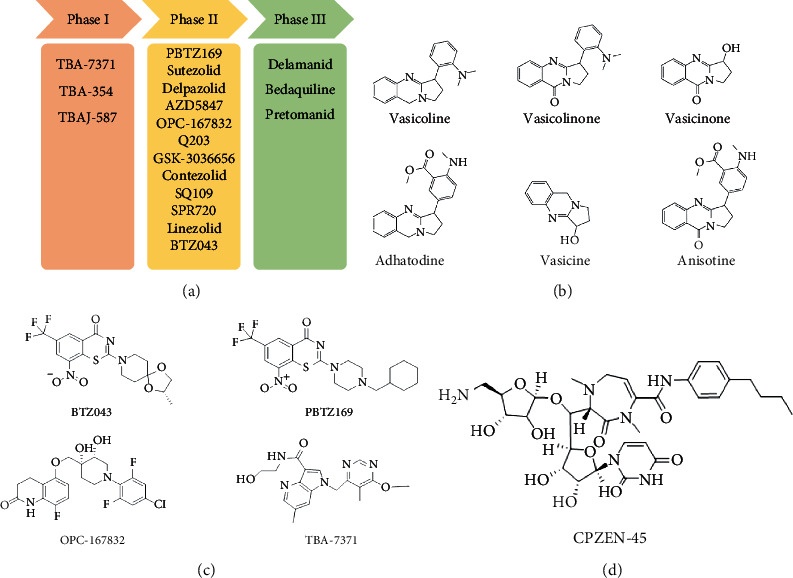
New anti-TB drugs in clinical trials (a), the chemical structures of vasicoline, vasicolinone, vasicinone, vasicine, adhatodine, and anisotine (b), the chemical structures of BTZ-043, PBTZ-169, OPC-167832, and TBA-7371 (c), and the chemical structure of CPZEN-45 (d).

Effective anti-TB regimens kill *Mtb*, improve the clinical symptoms of TB, and prevent the malignant development of the disease. The current standard treatment for drug-sensitive TB is a combination of a short-course chemotherapy regimen under direct supervision recommended by the WHO, which uses four first-line drugs [isoniazid (INH), RFP, ethambutol (EMB), and pyrazinamide (PZA)] in the first two months of development, followed by INH and RFP in the last four months of consolidation. The WHO TB guidelines in 2021 showed that this “short-course chemotherapy” has successfully cured 66 million patients with TB since 2000 [1]. However, the treatment of MDR-TB and XDR-TB is difficult and may have no significant effect on the persistent *Mtb*, because they need to be treated with more toxic and costlier second- and third-line drugs for a longer time, even up to two years, and usually with limited success. The primary causes of drug-resistant TB in clinical cases include gene mutations in drug targets or drug-activating enzymes, compensatory evolution, and the activation of efflux pumps [2, 3]. At present, there are limited varieties of anti-TB drugs in clinics, leaving clinicians with limited options. Therefore, there is an urgent need to develop new drugs with novel mechanisms to cure TB or shorten the treatment time for MDR-TB and provide effective support for TB control [4]. Here, we reviewed the 45 targets of drug action on *Mtb* and reported the corresponding newly developed drugs in recent years. These new drugs showed excellent anti-TB activity due to their action on different new targets, improved the cure rate of patients with MDR/XDR-TB, and significantly reduced the total mortality; however, some new drugs were more toxic than existing anti-TB drugs. Clearly, the rational and effective implementation of new drug regimens will overcome the “barrier” of drug-resistant TB and provide strong support for the goal of ending TB globally.  Table 1 shows the specific anti-TB targets, while Figure 1(a) presents the clinical trial stages of the new anti-TB drugs.

## 2. Cell Wall Biosynthesis

Cell wall biosynthesis offers several molecular targets because biosynthetic enzymes do not have homologs in the mammalian system. Traditional anti-TB first-line drugs (INH and EMB) and second-line drugs (cycloserine [CS] and ethionamide [ETH]) inhibit cell wall synthesis by acting on different targets.

### 2.1. Targeting Mycolic Acid Biosynthesis

The cell wall of *Mtb* contains a large amount of mycolic acid, which surrounds the peptidoglycan layer. Mycolic acids are considered the main virulence factor of *Mtb* because they make *Mtb* naturally resistant to most antibiotics [5]. The synthesis of the *Mtb* cell wall is mainly regulated by fatty acid synthases (FAS). FAS-I is also involved in the synthesis of fatty acids in eukaryotes, while FAS-II is unique to *Mtb* cells and is a target of anti-TB drugs. The biological enzymes enoyl-acyl carrier protein reductase (inhA) and *β*-ketoacyl-acyl carrier protein synthase III (FabH) are important targets of anti-TB drugs.

#### 2.1.1. Enoyl-acyl Carrier Protein Reductase

InhA is closely related to the extension of the fatty acid chain in the cell wall [6]. InhA is the only target of ETH and protionamide (PTH) and is one of the targets of INH. Mutations in *inhA* (*rv1484*) could induce *Mtb* resistance to ETH, PTH, and INH [7]. It has been confirmed that <10% INH-resistant *Mtb* clinical isolates are associated with mutations in *inhA* [7]. Several compounds with a diaryl ether structure and their derivatives have been identified as inhibitors of inhA, some of which showed activity against both drug-sensitive and drug-resistant *Mtb* [8, 9]. Triclosan, a diaryl ether derivative, is a potential anti-TB drug candidate because its polychlorinated molecular structure does not require any biological activation *in vivo* and directly targets inhA. However, these compounds have several limitations, such as undesirable bioavailability, limited solubility, and cytotoxicity [8]. *Mtb* has been reported to be resistant to triclosan, but the specific drug resistance mechanism is still unclear [10]. In addition, 3-nitropropanoic acid, gallic acid derivatives, pyrrolidone derivatives, tetrahydrofuran derivatives, 4-hydroxy-2-pyridone analogs, aryl amides, and other inhA inhibitors have been reported [9, 11–14].

#### 2.1.2. *β*-Ketoacyl-acyl Carrier Protein Synthase III

*β*-Ketoacyl-acyl carrier protein synthase III (FabH) is an important link between type I and type II FAS, and it catalyzes the condensation of malonyl-ACP with acyl-CoA to form *β*-ketoacyl-ACP [15]. *Mtb fabH* has no homologous proteins in humans. The six alkaloids (vasicoline, vasicolinone, vasicinone, vasicine, adhatodine, and anisotine, Figure 1(b)) extracted from the leaves of *Justicia adhatoda* and the selected synthetic small molecule compounds were found to have moderate activity against fabH [16, 17].

#### 2.1.3. Methoxy-mycolic Acid and Keto-mycolic Acid

Mycolic acid synthesized by *Mtb* is classified into three types according to positional modification: *α*-, keto-, and methoxy-mycolic acid [18]. Nitroimidazole compounds exert anti-TB activity by mainly targeting methoxy-mycolic acid.

Delamanid, a nitro-dihydro-imidazooxazole derivative, inhibits keto- and methoxy-mycolic acid synthesis and displays higher activity against *Mtb* than RFP, INH, EMB, and streptomycin (SM), without obvious cross-resistance with first-line anti-TB drugs [19]. However, delamanid monotherapy can rapidly produce delamanid-resistant *Mtb* strains.

In addition, PA-824 also acts on deazaflavin-dependent nitroreductase (Ddn). Ddn is mainly responsible for catalyzing the formation of lethal nitrogen atoms in cells by nitroimidazoles to cause bacterial cell lysis [20]. Phase II clinical trials showed that PA-824 disrupted the formation of mycolic acids and demonstrated potent activity against drug-sensitive and drug-resistant *Mtb*, including nonreplicating strains (https://www.croiconference.org/abstract/efficacy-bedaquiline-pretomanid-moxifloxacin-pza-bpamz-against-ds-mdr-tb/). In an anoxic environment, Ddn activates PA-824 and further generates lethal reactive nitrogen in *Mtb* cells, leading to nitric oxide (NO) release. In addition, as an NO donor, PA-824 can cause respiratory toxicity in bacterial cells and enhance the inherent killing mechanism of the innate immune system, resulting in bacterial death [21]. Under aerobic conditions, the methoxy and keto groups of mycolic acids in the cell wall of *Mtb* could be effectively inhibited by PA-824 to exert significant antibacterial activity [22].

TBA-354 has a narrow-spectrum bactericidal effect *in vitro* against replicating and nonreplicating *Mtb*. Its potency is similar to that of delamanid and greater than that of PA-824. TBA-354 has a higher bioavailability and a longer elimination half-life than other nitroimidazole drugs [23]. Unfortunately, the findings from the clinical trials showed that TBA-354 could result in mild signs of reversible neurotoxicity; therefore, Global TB Alliance discontinued this research [24].

### 2.2. Targeting Arabinogalactan Biosynthesis

#### 2.2.1. Decaprenylphosphoryl-*β*-D-ribose-2′-epimerase

Decaprenylphosphoryl-*β*-D-ribose-2′-epimerase (DprE) is a heterodimeric enzyme comprising DprE1 and DprE2 proteins. DprE1, a key enzyme in the cell wall biosynthesis of *Mtb*, was initially discovered as a target of benzothiazinones, which show potent activity against *Mtb* [25, 26]. Recently, four new drugs, namely, BTZ-043, PBTZ-169, OPC-167832, and TBA-7371 (Figure 1(c)), have been formally tested in the clinical trials (http://www.newtbdrugs.org/pipelines/clinical).

BTZ-043, the first discovered DprE1 inhibitor, exhibited more significant anti-TB activity than INH and EMB *in vitro* (minimum inhibitory concentration [MIC] 2.3 nM) and *in vivo* [27]. A phase I clinical trial of BTZ-043 has been completed (ClinicalTrials.gov identifier: NCT04874948), and a phase III clinical trial is recruiting now (ClinicalTrials.gov identifier: NCT04044001). Meanwhile, PBTZ-169 had lower cytotoxicity and better efficacy in a murine model than BTZ-043. The protonation of piperazine nitrogen makes it more soluble and improves its potency *in vivo* [28]. The phase II clinical trial of PBTZ-169 has been suspended owing to slow enrollment (ClinicalTrials.gov Identifier: NCT03334734).

OPC-167832, a newly synthesized carbostyril derivative, exerts potent inhibitory effects on both growing and intracellular *Mtb*. The MICs of OPC-167832 against *Mtb*, including MDR/XDR-*Mtb*, ranged from 0.24 to 2 ng/mL (https://www.newtbdrugs.org/pipeline/compound/opc-167832). *In vivo* experiments in mice showed that optimized regimens of OPC-167832 combined with delamanid have the potential to shorten the therapy course and significantly improve the prognosis of drug-sensitive TB and MDR-TB.

TBA-7371 is a noncovalent DprE1 inhibitor that has completed a phase I clinical trial. It inhibits DprE1 with an IC_50_ value of 10 nM and is active against *Mtb* with a MIC range of 0.78–3.12 *μ*M (https://www.newtbdrugs.org/). TBA-7371 could shorten the standard therapy course and has no cross-resistance to the current anti-TB drugs [29]. At present, a phase II clinical trial to assess the safety, early bactericidal activity, and pharmacokinetics of TBA-7371 is recruiting participants (ClinicalTrials.gov Identifier: NCT04176250).

#### 2.2.2. GlcNAc-1-P Transferase

GlcNAc-1-P transferase (WecA) is a phosphotransferase that catalyzes uridine diphosphate (UDP)-GlcNAc and decaprenyl-P to form decaprenyl-P-P-GlcNAc, which is further extended by rhamnosyl transferase to form decaprenyl-P-P-GlcNAc-rhamnose. WecA has been proposed as a target of the caprazamycin derivative CPZEN-45 (a *Streptomyces*-derived product, Figure 1(d)). CPZEN-45 showed excellent activity against both replicating and nonreplicating *Mtb* strains, with a MIC of 1.56 *μ*g/mL and 6.25 *μ*g/mL for *Mtb* H37Rv strain and MDR-*Mtb* strain, respectively. However, the development of an oral formulation for CPZEN-45 may not be feasible owing to its poor solubility and low bioavailability [30].

#### 2.2.3. TDP-6-deoxy-D-xylo-4-hexulose 3,5-Epimerase

**Figure 2:**
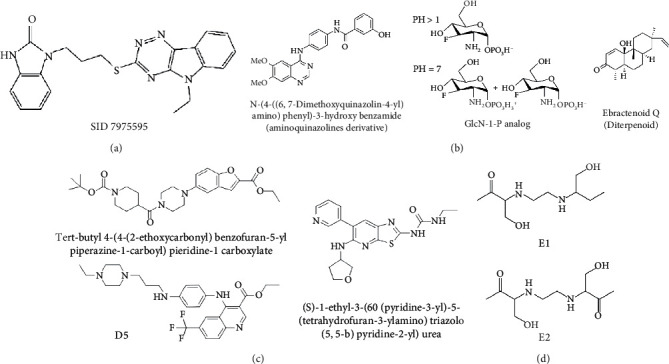
The chemical structures of SID 7975595 (a), the inhibitors of GlmU (b), the inhibitors of gyrB (c), and the inhibitors of E1 and E2 (d).

TDP-6-deoxy-D-*xylo*-4-hexulose 3,5-epimerase (RmlC), the rate-limiting enzyme in the production of thymidine diphospho-L-rhamnose, is responsible for converting dTDP-4-keto-6-deoxy-glucose into dTDP-4-keto-rhamnose [31]. Potential RmlC inhibitors with benzimidazolone structures were identified using high-throughput screening of 201,368 compounds. Nevertheless, *in vitro* experiments showed that most of these compounds displayed no significant anti-TB activities, except for compound SID 7975595 (Figure 2(a)), which exhibited an IC_50_ of 0.398 *μ*M on RmlC [32].

### 2.3. Targeting Peptidoglycan Biosynthesis

#### 2.3.1. Translocase I

Translocase I (murX), an essential enzyme for the growth of *Mtb*, converts UDP-N-acetylmuramyl-pentapeptide into prenyl-N-acetylmuramyl-pentapeptide (lipid I) in peptidoglycan biosynthesis [33]. MurX inhibitors (e.g., lead compound SQ641) rapidly killed *Mtb* during the growing stage. SQ641, a chemically modified nucleoside analog, killed *Mtb* faster than any existing anti-TB drugs and had a pronounced postantibiotic effect up to 55 h. However, a lipophilic decanoyl side chain in SQ641 likely contributes to its low solubility and poor oral absorption, resulting in modest intracellular activity against *Mtb* and poor *in vivo* activity [34]. Scientists have developed an SQ641 phospholipid nanoemulsion structure (SQ641-NE), which accommodates higher drug loading and is compatible with human use. SQ641-NE demonstrates better intracellular and *in vivo* activity and has synergistic interactions with the three first-line anti-TB drugs, INH, EMB, and PZA [35].

#### 2.3.2. Mycobactin

*Mtb* releases high-affinity siderophores, namely, mycobactin, which binds to iron more efficiently than host proteins [36]. MbtI, the product of *rv2386c* in *Mtb*, is the presumed isochorismate synthase that catalyzes the transformation of chorismate into salicylate and pyruvate in the first step of the mycobactin biosynthesis pathway. Being a chorismite-utilizing enzyme, mbtI does not exist in the host; therefore, mbtI inhibitors, including chromane-based derivatives and synthetic compounds, such as benzodihydropyranones, furans, and benzimidazole derivatives, are likely to be good therapeutic candidates for humans [37–39]. In addition, carbonyl and 7-hydroxyl groups are the necessary structures for these inhibitors.

MbtA, an adenylating enzyme encoded by *mbtA*, the initiating gene of mycobactin biosynthesis, catalyzes the synthesis of salicylic acid and ATP into salicyl adenylate. Nucleoside analogs, reported as mbtA inhibitors, exhibited potent anti-TB activity under iron-deficient conditions and had no obvious toxicity to mammals. However, these inhibitors possess unsatisfactory pharmacokinetic properties, including poor oral bioavailability, low oral exposure, and rapid clearance [40].

#### 2.3.3. Alanine Racemase and D-Ala-D-Ala Ligase

Among the existing second-line anti-TB drugs, CS is known to inhibit alanine racemase (alr) and D-Ala-D-Ala ligase (ddlA) with MIC values in the micromolar range. Alr is an essential enzyme for the racemization of L-alanine to D-alanine in most bacteria [41]. DdlA is a multidomain protein that binds two D-Ala fragments in the presence of ATP to form the precursor of the peptidoglycan D-Ala-D-Ala, which acts as a dipeptide donor in pentapeptide peptidoglycan biosynthesis [42]. Alr and ddlA are considered attractive drug targets owing to their essentiality in *Mtb* and the absence of a homolog in humans.

#### 2.3.4. N-Acetylglucosamine-1-phosphate Uridyltransferase

UDP-GlcNAc, an essential precursor of peptidoglycan and lipopolysaccharide in *Mtb*, is produced by a four-step biosynthetic pathway in the cytoplasm catalyzed by three enzymes [D-fructose-6-phosphate aminotransferase, phosphoglucosamine mutase, and N-acetylglucosamine-1-phosphate uridyltransferase (GlmU)] [43]. Deletion of GlmU in *Mtb* can lead to extensive perturbation of bacterial morphology and substantial reduction in cell wall thickness under normal and hypoxic conditions [44]. Three classes of compounds were identified as promising inhibitors of GlmU, namely, aminoquinazolines, GlcN-1-P analogs, and some diterpenoids extracted from the traditional Chinese medicines (Figure 2(b)) [45–47].

### 2.4. Targeting Other Targets in Cell Wall Biosynthesis

#### 2.4.1. Inositol-1-phosphate Synthase

Inositol is vital for the biogenesis of mycothiol, phosphatidylinositol, and glycosylphosphatidylinositol anchors linked to complex carbohydrates in *Mtb*. In the inositol biosynthesis pathway, glucose-6-phosphate is converted to inositol-1-phosphate through ino1. Ino1 is further dephosphorylated by inositol monophosphatase to produce inositol [48]. Ino1, encoded by *rv0046c*, is essential for *Mtb* growth. A previous study has shown that ino1 mutation in *Mtb* strains attenuated growth and reproduction in the normal medium [49]. Therefore, ino1 may be a rational target for the development of anti-TB drugs. However, it is necessary to consider the possible side effects of ino1 inhibitors due to the ubiquity of ino1 in eukaryotic cells.

#### 2.4.2. Methylerythritol Phosphate Pathway

Isopentenyl diphosphate (IPP) and its isomer dimethylallyl diphosphate (DMAPP) are crucial for the synthesis of arabinogalactan, peptidoglycan, and mycolic acid in the cell wall of *Mtb* [50]. It has been found that the mevalonate pathway and methylerythritol phosphate (MEP) pathway can be used in the biosynthesis of IPP and DMAPP. Most bacterial pathogens utilize the MEP pathway, whereas the mevalonate pathway is present in humans. A series of potent inhibitors of several enzymes in the MEP pathway, including fosmidomycin, thiazolyl pyrimidines, and natural products such as alkaloids, have been evaluated in the preliminary stage; therefore, further studies are needed on their pharmacological activities [50, 51].

## 3. Targeting Protein Biosynthesis and Breakdown

### 3.1. Targeting Protein Biosynthesis

In *Mtb*, the functional ribosomes are composed of 50S subunits (including 23S rRNA, 5S rRNA, and 35 proteins) and 30S subunits (including 16S rRNA and 22 proteins) [52]. Preliminary studies have demonstrated that most oxazolidinone compounds inhibit bacterial protein synthesis at the initial stage of transformation by binding to the V domain of 23S rRNA [53]. Certain other compounds act on the 50S ribosome subunit or leuRS to exhibit anti-TB activity.

#### 3.1.1. 50S Ribosome Subunits

AZD5847, an oxazolidinone derivative, blocks translation by binding with 50S ribosome subunits to inhibit protein synthesis. AZD5847 is a prodrug that is mainly activated into disodium phosphonate of AZD5847 *in vivo* to play an anti-TB role [54]. With regard to safety, AZD5847 showed adverse reactions of thrombocytopenia and hyperbilirubinemia when administered at high doses in phase II clinical studies [55].

#### 3.1.2. 23S rRNA

Sutezolid (PUN-100480), a novel oxazolidinone and a thiomorpholinyl analog of linezolid, significantly reduces the number of colony-forming units and acts by binding to the 23S ribosome [56]. Preliminary evidence showed that the activity of prodrug sutezolid in killing intracellular *Mtb* in patients was 10 times higher than that of its metabolite [57]. Phase I studies revealed that sutezolid was well tolerated at all dosages, was more active against MDR-*Mtb* isolates, and was less toxic than linezolid [58]. Phase II clinical trials showed that 14% of patients had a slight elevation in alanine aminotransferase levels [59]. At present, phase II and phase IV clinical trials are ongoing (ClinicalTrials.gov Identifiers: NCT03959566 and NCT03237182, respectively).

Delpazolid (LCB01-0371) is a second-generation oxazolidinone compound that contains a cyclic amidrazone. The compound had high aqueous solubility, good absorption, distribution, metabolism, excretion, and pharmacokinetic profiles, and low toxicity. In clinical trials, no obvious serious side effects were observed in any subjects, but mild diarrhea, dyspepsia, headache, nausea, and common adverse reactions were observed [60]. Currently, a phase II clinical trial was performed to evaluate the safety, efficacy, tolerability, and pharmacokinetics of delpazolid (combined with bedaquiline, delamanid, and moxifloxacin) in patients with pulmonary TB. This clinical trial is recruiting, and the result is not published (ClinicalTrials.gov Identifier: NCT04550832).

Contezolid (MRX-I), a new class of oxazolidinone protein inhibitors, is mainly used for the treatment of gram-positive bacteria. Eckburg et al. [61] reported that MRX-I had high bioavailability when taken with food and low drug accumulation after multidose administration. When a single dose was increased, the subjects showed good tolerance to MRX-I. Other studies have demonstrated that the anti-TB activity of MRX-I is comparable to that of linezolid, while MRX-I is less toxic in inhibiting bone marrow and monoamine oxidase [62, 63].

#### 3.1.3. Leucyl-tRNA Synthase

Leucyl-tRNA synthase (LeuRS) belongs to the class I aminoacyl-tRNA synthase subgroup and plays an important role in intracellular transport. GSK-3036656, a benzoxazole compound, exerts anti-TB effects by inhibiting *Mtb* LeuRS to block protein synthesis. The results of a phase I clinical trial showed that GSK3036656 was generally well tolerated after single and multiple doses; no serious adverse events were reported. In addition, GSK3036656 showed 2- to 3-fold accumulation when administered repeatedly, and the pharmacokinetic parameter experiments were not altered in the presence of food [64]. Recently, to evaluate the early bactericidal activity, safety, and tolerability of GSK3036656 in TB patients, a phase II clinical trial was conducted by GlaxoSmithKline in drug-sensitive pulmonary TB patients (ClinicalTrials.gov Identifier: NCT03557281); this clinical trial has been completed but the results are not yet available. Moreover, the results from another randomized, double-blind, placebo-controlled FTIH study showed that GSK3036656 is safe and generally well tolerated after single and multiple doses in healthy subjects (ClinicalTrials.gov Identifier: NCT03075410) [64].

### 3.2. Targeting Clp Proteases

Clp proteases, which are responsible for the degradation of abnormal proteins and nonfunctional proteins, are highly conserved intracellular proteolytic enzymes in prokaryotes and eukaryotes [65, 66]. The clp protease complex consists of a protease subunit (clpP) and an ATPase subunit (clpC or clpX) [67]. In contrast to the clp system of *Escherichia coli* with only one subunit, *Mtb* contains two coexpressed clpP subunits (clpP1 and clpP2) that function together with clpC1 and clpX, but both subunits have different substrate specificities [68]. It has been demonstrated that clp protease is a promising drug target [69]. To date, several compounds have been shown to target clp systems. For example, acyldepsipeptide antibiotics (ADEPs) and peptide boronates target clpP [70, 71], and four compounds, including lassomycin, cylomarin A, ecumicin, and rufomycin, targeting clpC1 ATPase showed potent activity against *Mtb* [72–75].

## 4. DNA-Related Enzymes

### 4.1. DNA Gyrase

DNA gyrase, which binds to DNA as a tetramer comprising two subunits A (gyrA) and two subunits B (gyrB), is the only type II topoisomerase in *Mtb*. The inhibition of the gene encoding DNA gyrase results in significant cell death because there are no viable alternative mechanisms for performing this function.

Fluoroquinolones (FQs), a class of antibiotics showing anti-TB activity, inhibit the supercoiling action of DNA gyrase by binding to the gyrA subunit and trapping the gyrase-DNA covalent complex. DC-159a is a newly synthesized broad-spectrum 8-methoxyfluoroquinolone that exhibits considerable inhibitory activity on the gyrA subunit of DNA gyrase in *Mtb* [76]. The MIC_90_ of DC-159a against drug-susceptible *Mtb* was 4 and 8 times higher than that of moxifloxacin and levofloxacin, respectively, with an MIC_90_ of 0.5 *μ*g/mL against MDR-*Mtb* isolates resistant to other FQs [77].

A novel GyrB inhibitor, SPR720 (a phosphate prodrug of SPR719), is currently in clinical development for the treatment of PTB. A randomized, double-blind, placebo-controlled, phase I clinical trial evaluated the safety, drug resistance, and pharmacokinetics of SPR720. The results showed that SPR720 was well tolerated orally, and the most common adverse events were mild to moderate gastrointestinal reactions and headache, which were shown to be dose-dependent (ClinicalTrials.gov Identifier: NCT03796910) [78]. Besides, active compounds with aromatic skeleton structures were identified through high-throughput screening, and they showed desirable gyrB inhibitory and anti-*Mtb* activities *in vitro* (Figure 2(c)) [79–81].

### 4.2. DNA Topoisomerase I

DNA topoisomerase I, encoded by *topA*, catalyzes the cleavage and binding of DNA strands by cleaving phosphodiester bonds on one strand of DNA and then religating the seal. It is a basic enzyme that maintains the biological growth activity of *Mtb* without the need for energy factors such as ATP [82]. Depletion of intracellular protein levels upon downregulation of *topA* expression led to the loss of *Mtb* viability.

Several inhibitors of DNA topoisomerase I have been identified. For example, boldine-derived alkaloids, dihydrobenzofuranyl urea, and amsacrine derivatives inhibited DNA topoisomerase I and showed moderate inhibitory effects on *Mtb* [82–84].

## 5. Energy Metabolism

### 5.1. ATP Synthase

ATP synthase provides the energy needed by *Mtb* during its life cycle (aerobic and hypoxic dormant stages). Bedaquiline (TMC207), sold under the brand name Sirturo, acts as an active substance against *Mtb* by inhibiting ATP synthase responsible for generating energy to *Mtb* cells.

Bedaquiline inhibits mycobacterial ATP synthase by binding to the C subunit and depletes cellular energy stores. It is highly selective for *Mtb* and has no obvious cytotoxicity to host cells. In addition, it has no cross-resistance with other anti-TB drugs and may be an important treatment option for patients with MDR-TB [85]. Bedaquiline has shown potent activity against drug-sensitive and MDR-*Mtb* strains with a MIC value of 0.03 *μ*g/mL, thus shortening the duration of treatment to 2–4 months [86]. Bedaquiline was approved for marketing by the Food and Drug Administration and was officially approved in China. In clinical trials, combining bedaquiline with other anti-TB drugs (e.g., FQs, clofazimine, PA-824, delamanid, and azithromycin) increased the risk of QTc prolongation in patients. As an indispensable drug in short-term MDR-TB regimens, the safety and efficacy of bedaquiline administrated with PA-824 and linezolid are being validated in phase III clinical trials (ClinicalTrials.gov Identifier: NCT 03086486). In addition, several clinical trials of bedaquiline are currently underway in different countries (https://www.clinicaltrials.gov/ct2/results?term=bedaquiline&cond=tuberculosis&Search=Apply&recrs=b&recrs=a&recrs=f&recrs=d&age_v=&gndr=&type=&rslt=).

At present, bedaquiline resistance has appeared. The drug resistance mechanisms can be summarized into three types: (1) as the target of bedaquiline, the mutation of *aptE* gene encoding the C subunit of ATP synthase weakened the binding force between bedaquiline and C subunit [87]; (2) the mutation of *Rv0678* gene encoding transcription suppressor of efferent pump MmpS5/MmpL5 will lead to upregulation of MmpS5/MmpL5 expression in efferent pump system and decrease intracellular drug concentration [88]; and (3) mutations in the *pepQ* gene may enhance efflux effect of the drug. Besides, bedaquiline and clofazimine showed complete cross-resistance to *Rv0678* and *pepQ* mutations [88, 89]. Therefore, the emergence of bedaquiline resistance will bring a new challenge to this novel drug treatment of TB.

### 5.2. Type-II NADH Dehydrogenase

Type-II NADH dehydrogenase (NDH-2) is a key enzyme in the respiratory chain for the growth and metabolism of *Mtb*. The absence of NDH-2 in mammalian mitochondria renders this enzyme an attractive target for antibiotic development [90]. Several compounds, such as phenothiazines, quinoline pyrimidines, quinazolones, and diphenyl iodine (DPI) analogs, have been found to be potent inhibitors of this enzyme [91–94].

### 5.3. Respiratory Cytochrome bc1 Complex

The respiratory cytochrome bc1 complex is responsible for catalyzing the transmission of electrons from hydroquinone to cytochrome, allowing protons to cross the plasma membrane in the respiratory chain of *Mtb*. Q203, an imidazopyridine amide, inhibits ATP synthesis by acting on the respiratory cytochrome bc1 complex. It inhibited MDR- and XDR-*Mtb* isolates at the nanomolar level and exhibited satisfactory anti-TB activity in mice infected with *Mtb* at a dose of <1 mg/kg. Q203 is effective against drug-resistant, MDR- and XDR-*Mtb* strains [95]. The result of phase І clinical trials demonstrated that Q203 was safe and well tolerated at different doses, and no significant adverse reactions were observed in the subjects (http://www.qurient.com/bbs/content.php?co_id=q203).

### 5.4. 1,4-Dihydroxy-2-naphthoyl CoA Synthase

Prokaryotes utilize menaquinone (vitamin K2) as a lipid-soluble redox cofactor in the electron transport chain. Menaquinone is mainly produced from mycolic acid, and the reaction is catalyzed by 1,4-dihydroxy-2-naphthoyl CoA synthase (menB); this biosynthesis pathway is absent in humans [96]. Therefore, mutation of the gene encoding menB hinders the transmission of electrons among membrane-bound protein complexes, and thus, it serves as a potential target for anti-TB drugs. It has been reported that 2-amino-4-oxo-4-phenylbutanoate derivatives containing a succinylbenzoate skeleton and some compounds with a benzoxazine skeleton structure exhibited good inhibitory effects on both replicating and nonreplicating *Mtb* by inhibiting menB [97, 98].

## 6. Lipid Metabolism

### 6.1. Fatty Acid Desaturase

Fatty acid desaturase in *Mtb* catalyzes the oxidation of alkyl-saturated fatty acids to produce alkyl-unsaturated fatty acids by introducing two *cis*-double bonds at the proximal or distal end [99]. Three potential aerobic desaturases (encoded by *desA1*, *desA2*, and *desA3*) were identified through the whole genome analysis of *Mtb* [100]. DesA3 (Rv3229c) is a membrane enzyme with stearyl-CoA9-desaturase (*Δ*^9^-stearyl desaturase) activity that produces oleic acid, which is an essential component of mycobacterial membrane phospholipids and triglycerides. Thiocarlide, an effective anti-TB drug developed in the 1970s against a range of MDR-TB strains, causes a decrease in oleic acid synthesis by inhibiting desA3 and in stearic acid synthesis of *Mtb*, eventually resulting in cell death [101].

### 6.2. Pantothenate Synthetase

Pantothenic acid, also known as vitamin B5, is an important precursor of coenzyme A (CoA) and ACP and plays a decisive role in the energy exchange of *Mtb*. In the process of pantothenic acid biosynthesis, four pantothenate synthetases (pan) (B, C, D, and E) are involved. Of these, panC is the rate-limiting enzyme in the synthesis process, which catalyzes the final step of the reaction. Some compounds containing aromatic heterocyclic skeletons have been identified as inhibitors of panC of *Mtb* [102]. However, further modifications need to be considered to improve potency and selectivity based on the structural characteristics of the active site of the enzyme.

## 7. Signal Transduction Pathways

### 7.1. Shikimic Acid Pathway

**Figure 3:**
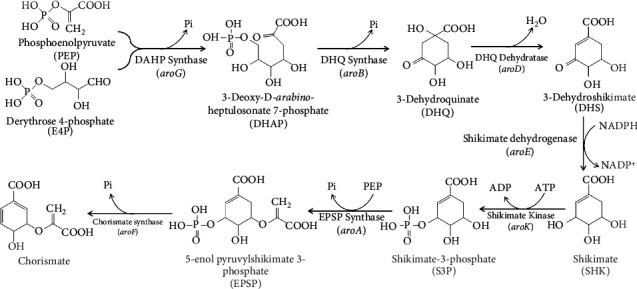
The shikimate pathway of *Mycobacterium tuberculosis*.

In the shikimate pathway, erythrose-4-phosphate is converted to the final product chorismate through seven enzymatic steps (Figure 3) [103]. It is thought to inhibit the growth of *Mtb* by inhibiting the activities of seven related enzymes involved. Shikimate kinase (SK), encoded by *arok*, is a key enzyme that converts shikimic acid to shikimic acid-3-phosphate catalyzed by ATP.

Dicyclic sesquiterpenes, which are natural products linked to quinones and synthetic small molecular compounds 3-nitrobenzyl derivatives, are promising SK inhibitors. The sesquiterpene derivative ilimaquinone (IQ) showed time-dependent inhibitory activity against *Mtb* shikimate kinase (MSK). Besides, 3-nitrobenzyl derivatives and pyrazole derivatives compounds also showed strong inhibitory activity against MSK [104, 105].

## 8. Other Targets

### 8.1. Filamenting Temperature-Sensitive Protein Z (FtsZ)

FtsZ, a bacterial tubulin homolog, is a significant cell division protein that attaches to the membrane of the bacterial center and forms a cell dynamic ring called the Z ring. Inactivation of ftsZ or alteration of ftsZ protein can inhibit the formation of the Z ring and septum, which in turn affects cell division [106].

Several ftsZ inhibitors have been found to be effective against *Mtb*, including natural products containing curcumin, coumarins, berberine, and resveratrol, small-molecule compounds comprising bisindole methane derivatives, benzamides, taxanes, and rhodamine derivatives, and some polypeptides and nucleic acids [107–110]. Most of these compounds have been tested *in vitro*. Some tricyclic substituted benzimidazoles have been evaluated *in vivo* in mice, and they showed potent anti-*Mtb* activity [111].

### 8.2. Mycobacterial Membrane Protein Large Proteins

Resistance-nodulation-division (RND) is a ubiquitous family of efflux pumps that may contribute to the recognition and transport of a great diversity of cationic, anionic, or neutral compounds in bacteria [112, 113]. Among the RND superfamily of transporters, mmpL proteins play an important role in the elaboration of the cell envelope of mycobacteria. The genes encoding for mmpL proteins are associated with gene clusters involved in the synthesis of cell wall-associated glycolipids [114]. The anti-TB lead compound BM212 and the new drug SQ109 target mmpL3 [115].

The potent compound BM212, a 1,5-diarylpyrrole derivative, showed MIC values ranging from 0.7 to 1.5 *μ*g/mL against several *Mtb* strains. However, owing to its poor bioavailability and severe toxicity, BM212 was not subjected to clinical trials [115].

SQ109, an ethylenediamine compound optimized from a library based on EMB, is currently the only candidate drug for use in clinical research. It was considered a potential candidate owing to its submicromolar MIC values (0.78-2 *μ*M) and low cytotoxicity. SQ109 showed improved activity on intracellular *Mtb* compared to EMB and at a similar level to INH. Several studies have reported that in addition to inhibiting *Mtb* cell growth, SQ109 also acts on the growth of other bacteria, fungi, and malarial parasites, all of which lack the common functional target point mmpL3 orthologs [116–118]. Therefore, it can be presumed that mmpL3 is not the only target of SQ109.

### 8.3. Ser/Thr Protein Kinase

The presence of several STPKs suggests that protein phosphorylation plays a central role in regulating various biological functions, ranging from environmental adaptive responses to bacterial pathogenicity. Among the 11 *Mtb* STPKs, only protein kinase A (pknA), pknB, pknG, and serine/threonine phosphatase are essential for intracellular survival of *Mtb* cells [119]. The anti-TB efficacy of pknA inhibitors was not significant. The IC_50_ values of many compounds that inhibited pknB were at the micromolar level; however, these compounds had no effect on *Mtb* [120–122].

### 8.4. Alkyl Hydroperoxidases

Although the alkyl hydroperoxidases ahpD and ahpC of *Mtb* have no sequence homology, they are regulated by the same promoter. AhpC provides hydrogen to protect *Mtb* from H_2_O_2_ or other oxidants, while ahpD reduces ahpC in an oxidized state, thereby ensuring that ahpC can continue to catalyze the reactions and guarantee recycling. On the other hand, ahpD exhibits alkylhydroperoxidase activity, and the highly oxidative environment in macrophages prevents ahpD from providing antioxidant protection to the internally retained *Mtb*. Therefore, the catalytic reaction of ahpC can be blocked by inhibiting ahpD activity in the antioxidant system of *Mtb*. In this case, *Mtb* is no longer protected by alkylhydroperoxidase, resulting in cell lysis [123]. These features make ahpD a potentially attractive target for anti-TB drugs.

### 8.5. Arylamine N-Acetyltransferase

NATs constitute a major family of enzymes that acetylate arylamines and hydrazines using acetyl-CoA as an acetyl donor [124]. A NAT isoenzyme in humans (known as human NAT2) is responsible for the acetylation and inactivation of INH. If INH is acetylated by NAT2, the product acetylisoniazid cannot be oxidized to its active form (isonicotinic acid) by katG. Therefore, the bioavailability of INH can be improved by effectively inhibiting the activity of NATs to kill *Mtb*.

### 8.6. Diterpene Cyclase and Diterpene Synthase

Diterpene cyclase and diterpene synthase, encoded by *rv3377c* and *rv3378c*, respectively, produce diterpenoids of tuberculosinols in the cell membrane of *Mtb*, which ensures the pathogenicity and virulence of *Mtb* [125]. Rv3377c cyclizes bicyclization and rearrangement of (E, E, E)-geranylgeranyl diphosphate to halimadienyl-diphosphate (HPP). Meanwhile, Rv3378c hydrolyzes HPP to the novel tricyclic diterpene edaxadiene, which directly inhibits phagosomal maturation *in vitro* [126–128]. Diterpenoid and triterpenoid acids, including isosteviol and betulinic, oleanolic, and ursolic acids, as well as binuclear isosteviol derivatives, showed moderate anti-TB activity by competitively binding to the active site of diterpene synthase.

### 8.7. Isocitrate Lyase (ICL)

ICL is a pivotal enzyme that catalyzes the first step of the glyoxylate cycle and has been demonstrated to be essential for *Mtb* survival against macrophages [129]. Studies have shown that the persistence of *Mtb* disappears after the deletion of the ICL gene. The 3-nitropropionamide derivatives, which are synthetic compounds, showed potent antimycobacterial activity with an IC_50_ value of 0.1 *μ*M [130, 131].

### 8.8. Redox-Related Enzymes

Cytochrome P450 enzymes, belonging to the monooxygenase system, are mainly distributed in the endoplasmic reticulum and mitochondrial inner membrane of eukaryotic cells and are found freely in the cytoplasm of prokaryotic cells. Antifungal azoles, such as fluconazole and itraconazole, inhibit cytochrome P450-dependent enzymes. Studies have shown that econazole and clotrimazole are most effective against *Mycobacterium smegmatis* (MIC values <0.2 and 0.3 *μ*M, respectively). Moreover, they are superior inhibitors of mycobacterial growth than RFP and INH, suggesting that CYP51 might be a potential target for azole drugs to exert anti-TB activities [132].

### 8.9. Mycothiol

Mycothiol (MSH) protects *Mtb* from oxidants and cytotoxins. The biosynthesis of MSH is a multistep process that four enzymatic reactions catalyzed by mshA, mshB, mshC, and mshD. Both mshB and mshD are not essential for the growth of *Mtb*, while the knockout of *mshA* and *mshC* could cause fatal damage to the growth of *Mtb* [133]. Three classes of compounds comprising the adenosine derivatives 5-*O*-[*N*-(L-cysteinyl) sulfamonyl] adenosine, NTF1836, and dequalinium chloride, were identified as promising inhibitors of mshC [134–136].

### 8.10. 4′-Phosphopantetheinyl Transferases

Phosphopantetheinyl transferases (PptTases) play a major role in activating fatty acids, polyketides, and nonribosomal peptide synthases, which endow *Mtb* with its unique ability to produce an impressive variety of lipids with unusual structures. PptT, one of two PptTases produced by *Mtb*, is encoded by *rv2794c* and is responsible for the activation of Pks13 enzyme and various type-І Pks required for the formation of mycolic acids and lipid virulence factors in mycobacteria [137]. Amidino-urea 1-[(2,6-diethylphenyl)-3-N-ethylcarbamimodoyl]urea, called “8918”, effectively killed *Mtb*, including drug-resistant clinical strains [138]. The “8918” showed an MIC_90_ of 3.1 *μ*M and 0.56–3.0 *μ*M against *Mtb* laboratory strain and 29 *Mtb* clinical strains, respectively [138]. However, the short half-life of “8918” leads to rapid microsomal metabolism, resulting in insufficient time to eliminate *Mtb in vivo*.

### 8.11. MptpA and mptpB

*Mtb* encodes two protein tyrosine phosphatases, mptpA and mptpB, which act as key virulence factors and are secreted during infection and are important for the entry and survival of *Mtb* in the host cell. At present, four classes of compounds have been identified as promising inhibitors, namely, chalcones, aryldifluoromethylphosphonic acids, cyclic peptides, and halogen-containing aromatic compounds [139–142]. The IC_50_ values of compounds with the best anti-mptpA and anti-mptpB activities were 0.16 and 0.038 nM, respectively [142].

### 8.12. Biofilm

Biofilm is an organized colony of bacteria surrounded by large extracellular molecules of bacteria attached to the surface of living or inanimate objects. *Mtb* forms biofilms in an anaerobic environment and is highly resistant to anti-TB drugs. Although the mechanism of biofilm formation of *Mtb* is not clear, the formation process of it can be divided into reversible adhesion and irreversible adhesion [143]. The formation mechanism of *Mtb* biofilm is similar to that of other bacterial biofilm. The biofilm maturation process of *Mtb* includes four steps, such as attachment, sessile growth, biofilm maturation, and dispersal [144]. It has been reported that compound C10 could prevent the formation of a pellicle biofilm to a large extent, making *Mtb* more susceptible to antibiotics [145]. C10 in conjunction with INH may restore the efficacy of INH in patients with INH-resistant TB and increase the efficacy of antibiotics in killing *Mtb*, including those of INH-sensitive strains.

### 8.13. Type VII Secretion System

The pathogenicity of *Mtb* is related to its type VII secretion system, which secretes a large number of effector proteins to resist the host's immune defense and promote *Mtb* infection. The type VII secretion system of *Mtb* consists of five members: ESAT-6 secretion systems (ESX) 1 to 5, among which ESX-1 is the most widely studied. The ESX-1 gene cluster is located in the RD1 region, which is not included in the BCG strain, suggesting that ESX-1 plays a critical role in *Mtb* virulence [146, 147]. The pathogenicity of ESX-1 deficient *Mtb* mutants is highly attenuated [148, 149]. Several ESX-1 inhibitors have been successfully identified and may be promising anti-TB drugs [150, 151]. Recent studies have shown that IMB-BZ can specifically inhibit the secretion of CFP-10 to reduce virulence, which will significantly reduce the survival of *mycobacteria* in the intracellular and *in vivo* [152]. In addition, the inhibitors of protein synthesis (chloramphenicol and kanamycin) and protein degradation (lassomycin and bortezomib) can specifically block ESX-1 secretion activity in *Mtb* and reduce the growth of *Mtb* by 50% or less [153]. Moreover, subinhibitory concentrations of chloramphenicol can specifically attenuate ESX-1-mediated *Mtb* virulence in macrophages [153]. Taken together, targeting ESX-1 may promote the development of new TB drugs, and these potential inhibitors still need to be further studied.

## 9. Problems and Prospects

At present, steady progress has been made in the research and development of new anti-TB drugs, many promising drug targets have been identified, and many new and different action mechanisms of the leading compounds, candidate drugs, and anti-TB drugs have been developed. However, there are still problems and challenges.

### 9.1. Elucidate the Biological Characteristics and Pathogenesis of *Mtb*

The genes, proteins, and pathways involved in the growth and metabolism of *Mtb* and their pathogenesis are undoubtedly the strategies for discovering new drug targets. Based on the physiological characteristics of bacterial membranes, Chen et al. [154] presumed that *Mtb* cell membranes could be used as a feasible target for blocking bacterial persistence and sustained survival. All biological organisms, regardless of their replication state, need to rely on functionally and structurally complete cell membranes to survive. Bacterial cell membranes are disrupted when numerous physiological metabolic targets and their transduction pathways are inhibited. However, at present, the selection of targets in the membrane is a major obstacle to the development of cell membrane inhibitors. Lipophilic drugs may cause toxic effects on mammalian cell membranes, resulting in irreversible damage to host cells [154]. These challenges notwithstanding, selective targeting of *Mycobacterium* cell membranes offers an opportunity to solve the problems of existing anti-TB drugs.

The pathogenicity of *Mtb* is related to the inflammatory response caused by the mass reproduction of *Mtb* in tissue cells, the components of the bacteria and cell walls, the toxicity of metabolites, and the anti-TB immune response of the body. Although there is abundant evidence that lipids may be the main pathogenic agent of *Mtb*, all pathogenic substances and their pathogenic mechanisms have not been fully elucidated. At least five different cell wall glycolipids are released by *Mtb* into the body's macrophages. The release of these components may affect physiological processes in and between cells. Therefore, lipid studies are essential to elucidate the pathogenesis of *Mtb* and to identify effective targets.

### 9.2. Strengthen the Research of Anti-TB Drug Targets

Identifying drug targets is the key to the research and development of new anti-TB drugs, and the ideal anti-TB drug target obtained should meet the following conditions: (1) the target should be the molecule necessary for the growth and metabolism of *Mtb*; once inactivated, it will lead to death or the lack of retention ability of *Mtb*; (2) the target does not rapidly produce drug resistance or bypass pathway; (3) there is no homology between the target and human molecules; and (4) the target should be different from other compounds already discovered. Some new action mechanisms and action targets, such as the biological characteristics of retentive *Mtb*, tissue liquefaction and cavity formation, and the host immune mechanism of latent tuberculosis infection, have also become research hotspots.

If the latent mechanism of *Mtb* could be elucidated and identify the targets clearing latent *Mtb*, it will greatly shorten the course of TB treatment and reduce the production of drug-resistant *Mtb*. For instance, the glyoxylate pathway is involved in the pathogenicity of a variety of pathogens, which can play a broad-spectrum antibacterial role as a drug target.

In addition, we need to expand the research ideas to focus on those proteins related to TB in the host, in which increasing or reducing the expression of these proteins can destroy the survival environment of *Mtb* and enhance the resistance of the body. The latent mechanism underlying *Mtb* in macrophages can be elucidated to find a target to break its immune escape in macrophages to promote the clearance of latent *Mtb*.

One of the traditional methods of drug target discovery is to identify the target through molecular pharmacology studies of drugs with significant pharmacological effects. Although lead compounds with pharmacological effects can be observed through animal experiments, disadvantages including duration of the study, high cost, and low efficiency are noted. Bioinformatics, including proteomics, genomics, gene function prediction, and data mining, have been used in drug development processes in recent years. A study established a characteristic model of cell body-related genes and identified 127 potential cell wall-related genes through bioinformatics analysis of *Mtb*, which provided strong support for the discovery of drug targets in the future [155]. In addition, a recent study confirmed that arabinosyltransferase C, which is involved in the cell wall synthesis of *Mtb*, was a potential anti-TB target from the insight of molecular docking and found that E1 and E2 (Figure 2(d)) with binding affinities of −5.77 kal/mol and−5.13 kal/mol, respectively, could be potential inhibitors of arabinosyltransferase C [156]. The combination of biotechnology, such as gene sequencing, connectivity mapping, and molecular docking, has become a new strategy for identifying drug targets for different diseases. Simulated binding with small molecular compounds using a structure simulation technique may quickly screen the inhibitors of the target from a compound library. Moreover, the application of gene manipulation techniques, such as gene knockout and gene transfer, makes the identification of drug targets easier. Therefore, the analyses of drug targets from the perspective of biology and statistics have broken through the bottleneck of traditional cytological or molecular screening methods and have greatly improved the screening speed and efficiency of new anti-TB drug targets.

### 9.3. In-Depth Study and Modification of Lead Compounds with Anti-TB Activity

It is well known that target inhibitors are identified mainly through high-throughput screening, which often ignores the inhibitory activity of compounds against *Mtb* and the adverse reactions to the host itself, leading to the slow discovery of effective lead compounds. A large number of novel lead compounds with anti-TB activity have been identified using high-throughput screening methods, but the mechanism of action of a few new drugs remains unclear. Studying the active molecular groups and mechanism of action of the lead compounds can modify the lead compounds to obtain more active anti-TB drugs. In recent years, after an in-depth study of the structure and anti-TB mechanism of bedaquiline, which still has safety concerns including QTc prolongation and hepatotoxicity, scientists have found that replacing the naphthalene C units with different heterocycles could confirm its efficacy and reduce its side effects, among which the most classical structural modification compound is TBAJ-587 [157, 158]. Recent animal studies have shown that TBAJ-587, with additional improved properties, has better anti-TB activity, safety, and pharmacokinetic properties and a lower predicted clinical dose than bedaquiline (https://www.newtbdrugs.org/pipeline/compound/tbaj-587-diarylquinoline, https://www.tballiance.org/portfolio/compound/tbaj-587-diarylquinoline). Currently, a randomized, double-blind, placebo-controlled phase I clinical trial is recruiting healthy adults to evaluate the safety, tolerability, and PK of TBAJ-587 (NCT04890535).

### 9.4. Identify Anti-TB Activity from Existing Antibiotics or Natural Products

There is no doubt that the discovery of new chemical scaffolds with novel mechanisms of action is risky. Therefore, it is an effective strategy to identify the anti-TB activity of existing antibiotics or optimize their structure to find new anti-TB drugs. Linezolid is a synthetic oxazolidinone antibiotic approved by the United States Food and Drug Administration in 2000 to treat infections caused by gram-positive cocci. In recent years, linezolid has been shown to be effective in the clinical treatment of MDR-TB [159]. Due to the lack of drugs for treating drug-resistant TB, a combination therapy strategy containing linezolid has been reported to be a promising option for MDR-TB and XDR-TB treatment [160]. At present, owing to the serious side effects of longer-term use, including bone marrow suppression and optic and peripheral neuropathy, limits the clinical application of linezolid [161]. If this concern is amended through structural modification, linezolid analogs could be developed as new anti-TB drugs in the future. Metronidazole, a class of nitroimidazole antibiotics, has been widely used to treat local infections caused by anaerobic bacteria in clinical settings and has entered the clinical trial stage of anti-TB in recent years (https://www.clinicaltrials.gov/ct2/results?cond=tuberculosis&term=metronidazole&cntry=&state=&city=&dist=). The approved delamanid and PA-824 were derived from the metronidazole scaffold through different chemical structure modifications. The advantage of this strategy is that there is a large amount of pharmacodynamics and human safety data available that can be quickly entered into clinical trials, but the number of antibiotics that can be reevaluated is limited.

Although a large number of natural products have emerged with anti-TB activity, few natural products or derivatives have been developed into drugs since the discovery of RFP in 1968. There were few unmodified natural products that could exert the drug activity directly, all of which need to be modified by introducing chemical groups. CPZEN-45 and capuramycins, natural product derivatives with detailed synthetic routes discovered in recent years, were all suitable for the treatment of TB. Nevertheless, it is still necessary to develop more unknown natural products to expand the pipeline of anti-TB drugs. Scientists have isolated more than 170 compounds with anti-TB properties from marine organisms, of which 10 had strong activities and potential for further development [162]. In addition, traditional Chinese medicine is a promising source for the development of anti-TB lead compounds. For example, cordycepin, an efficient component of *Cordyceps* spp., kills *Mtb* by hijacking bacterial adenosine kinase [163]. An active compound isolated from *Arisaema sinii* Krause could inhibit biofilm formation of *Mtb* [164]. However, these studies are limited to *in vitro* experimental studies, and there are few reports on further animal experiments and clinical trials. Therefore, the development of traditional Chinese medicine against TB is relatively slow.

### 9.5. Computational Technologies for Screening and Developing Novel Anti-TB Drugs

In recent years, with the development of bioinformatics, a growing number of computational technologies have been used for drug design and development. Compared with the traditional drug screening methods, the emerging computational approaches for drug screening greatly improve the chance of determining effective drug molecules and save time and effort. For example, Puhl et al. developed several computational screening approaches based on the identified crystal structure of DG167 in KasA protein which is involved in mycolic acids synthesis and identified several drug molecules that bind effectively to KasA protein [165]. Almeleebia et al. selected 224,205 compounds and screened the catalytic site of *Mtb* proteasome by the computational approach and then docked the top hit compounds with the *Mtb* proteasome molecule, discovering the interaction mechanism between these compounds and the proteasome and successfully identifying several *Mtb* proteasome inhibitors [166]. The effective combination of machine learning, artificial intelligence, CRIS, and other technologies will greatly save time and reduce the cost, providing a powerful weapon in the fight against TB in the future [167].

## 10. Conclusion

In summary, there are currently more than 15 kinds of anti-TB drugs in clinical trials, but only bedaquiline and delamanid have been listed with a new target and structure as of yet. Screening more anti-TB candidates is urgently needed to develop more effective and safer anti-TB drugs, especially against MDR-TB and XDR-TB. Cell wall synthesis, ATP synthesis, protein synthesis, DNA synthesis, and other signal transduction pathways of *Mtb* mentioned in this review have been considered the research hotspots of anti-TB drug development in recent years. Targeting persistent *Mtb* in infected host cells has become a focus of research in this field. In the future, we should find more reasonable and effective targets to develop new drug candidates with high activity and fewer adverse reactions, and the research and development of anti-TB drugs still have a long way to go.

## Figures and Tables

**Table 1:** Summary of targets of anti-TB drugs.

Action targets		Gene Bank	Human homologue	New drugs/lead compounds	Structure	Mechanism of action	NCT number	Phase	Notes	References
Cell wall biosynthesis	InhA	*rv1484*	None	Diaryl ether compounds	—	—	—	—	Undesirable bioavailability, limited solubility, and certain cytotoxicity	[6, 8, 9, 11, 12, 168]
	FabH	*rv0533c*	None	Alkaloids and small molecule compounds	—	—	—	—	*In vitro* experiments	[15–17]
	Methoxy-keto-mycolic acid	*—*	None	Delamanid	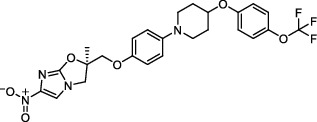	Inhibit the mycolic acid biosynthesis of *Mtb* cell wall	NCT03828201	II	Not yet recruiting	[18–24, 169]
							NCT03959566	II	Recruiting	
							NCT04550832	II	Recruiting	
							NCT05007821	II	Recruiting	
							NCT04518228	IV	Recruiting	
				Pretomanid (PA-824)	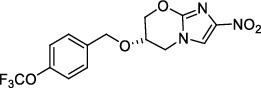		NCT04179500	II	Recruiting	
							NCT05040126	III	Recruiting	
							NCT04207112	II, III	Recruiting	
				TBA-354	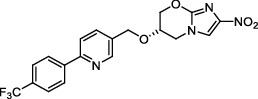		NCT02606214	I	Terminated (mild reversible neurotoxicity)	
	DprE1	*rv3790*	None	BTZ-043	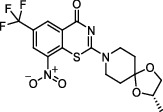	Inhibit the arabinogalactan biosynthesis of *Mtb* cell wall	NCT04874948	I	Completed	[4, 25–29, 170]
							NCT04044001	I, II	Recruiting	
				PBTZ-169	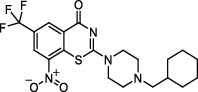		NCT03334734	II	Terminated (very slow enrollment)	
							NCT03776500	I	Completed	
				OPC-167832	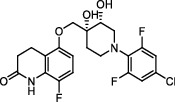		NCT03678688	I, II	Recruiting	
				TBA-7371	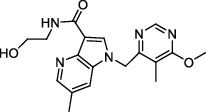		NCT03199339	I	Completed	
							NCT04176250	II	Recruiting	
	WecA	*rv1302*	None	CPZEN-45	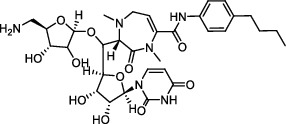		—	Preclinical study	Poor solubility and potentially low bioavailability	[30]
	RmlC	*rv3465*	None	Benzimidazolone derivatives	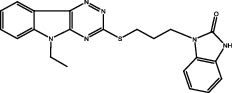		—	—	Mild antitubercular activity	[31, 32]
	MurX	*rv2156c*	None	SQ641	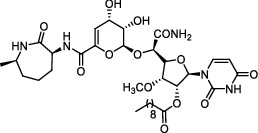	Inhibit the peptidoglycan biosynthesis of *Mtb* cell wall	—	Preclinical study	Low solubility and low bioavailability	[33–35]
	MbtI	*rv2386c*	None	Natural products and small molecule compounds	—		—	—	Carbonyl and 7-hydroxyl groups are the necessary structures of inhibitors	[36–38, 40, 171]
	MbtA	*rv2384*		Nucleic acid analogues					Short half-life and low bioavailability	
	Alr and DdlA	*rv3423c* *rv2981c*	None	—	—		—	—	Research needs to be further	[41, 42]
	GlmU	*rv1018c*	None	Natural products and small molecule compounds	—		—	—	*In vitro* experiments	[43–46, 172]
	Ino1	*rv0046c*	Yes	—	—	—	—	—	Inhibitors rarely reported so far	[48, 49, 100]
	MEP pathway	*—*	None	Alkaloids and small molecule compounds	—	—	—	—	Research needs to be further	[50, 51]
Protein biosynthesis and breakdown	50s ribosomal subunit	*—*	None	AZD-5847	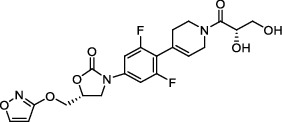	Inhibit the proteins biosynthesis of *Mtb*	NCT01516203	II	Completed	[54, 55]
	23s rRNA	*—*	None	Sutezolid	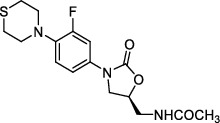		NCT03959566	II	Recruiting	[57–63]
							NCT03237182	IV	Recruiting	
				Delpazolid	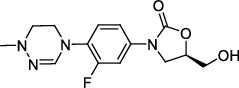		NCT04550832	II	Recruiting	
							NCT04939779	I	Completed	
				Contezolid	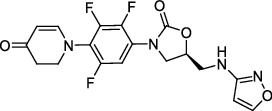		NCT03747497	II	Completed	
	LeuRS	*—*	Yes	GSK-3036656	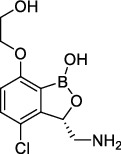		NCT03557281	II	Completed	[64]
	ClpP1 ClpP2	*rv2461c* *rv2460c*	Yes	Acyldepsipeptide antibiotics (ADEPs) and cyclomarinA	—	—	—	—	Research needs to be further	[69]
DNA related enzymes	GyrA *rv0006*		None	DC-159a	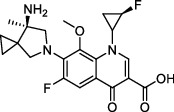	Inhibit the activity of DNA gyrase, blocks DNA replication and transcription to lead *Mtb* death	—	Preclinical study	*Mtb* is more resistant to DC-159a	[77]
	GyrB *rv0005*		None	SPR720	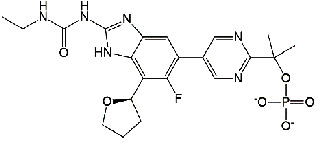			I	Completed	[78]
	topA	*rv3646c*	Yes	Boldine-derived alkaloids	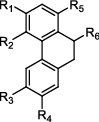	—	—	Screening and structural optimization	Inhibition activity at *μ*M level	[82–84]
Energy metabolism	ATP synthase	*—*	Yes	Bedaquiline	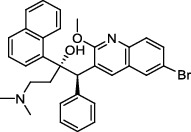	Inhibit the ATP synthase of *Mtb* to block the energy supply	NCT04630145	II and III	Recruiting	[85, 86]
							NCT04207112	II and III	Recruiting	
	NADH-2	*rv1854c*	None	Phenothiazines, quinoline pyrimidines, quinazolones, and DPI analogues	—	—	—	—	More serious side effects	[90–94, 173]
	Respiratory cytochrome bc1 complex	*—*	Yes	Q203	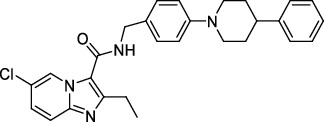	Inhibit the synthesis of ATP by acting on the respiratory cytochrome bc1 complex	NCT03563599	II	Completed	https://www.clinicaltrials.gov/ct2/show/NCT03563599?cond = Q203&draw =2&rank =1
	MenB	*rv0548c*	None	Succinyl benzoate derivatives and benzoxazine derivatives	—	—	—	—	*In vitro* experiments	[96–98]
Lipid metabolism	Fatty acid desaturase (DesA3)	*rv3229c*	Yes	Thiocarlide	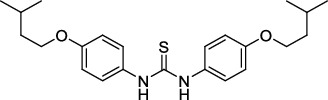	Inhibit desA3 to decrease oleic acid and stearic acid synthesis of *Mtb*	—	—	Require structure optimization	[99, 101, 174] [100]
	Pantothenate synthetase (PanC)	*rv3602c*	None	Small molecule compounds	—	—	—	—	*In vitro* experiments	[102]
Signal transduction pathway	Shikimate pathway	*rv2539c*	None	Natural products and small molecule compounds	—	—	—	—	*In vitro* experiments	[103, 104, 175]
Other targets	FtsZ	*rv2150c*	None	Natural products and small molecule compounds	—	—	—	—	*In vitro* experiments	[106–109, 111]
	MmpL	*rv0206c*	None	BM212	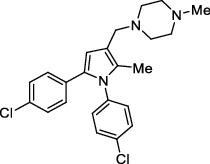	Inhibit membrane proteins mmpL of *Mtb*	—	Preclinical study	Poor pharmacokinetics and more severe toxic reactions	[112–118, 176]
				SQ109	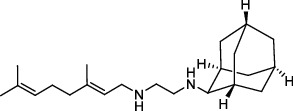		NCT01785186	II	Completed	
	STPK	*—*	Yes	—	—	—	—	—	Antituberculosis activity of inhibitors is not significant	[119–122]
	AhpD	*rv2429*	None	—	—	—	—	—	Inhibitors rarely reported so far	[123]
	NATs	*rv3566c*	Yes	—	—	—	—	—	Inhibitors rarely reported so far	[124]
	Diterpene cyclase	*rv3377c*	None	Diterpenoid and triterpenoid acids	—	—	—	—	Research needs to be further	[125–128]
	Tuberculosinol phosphatase	*rv3378c*								
	ICL	*rv0467*	None	Natural products and synthetic compounds	—	—	—	—	In laboratory research	[129–131]
	Redox-related enzymes (cytochrome P450 enzymes)	*—*	Yes	Azole antifungals	—	—	—	—	Prevent the synthesis of ergosterol from fungal cell membrane	[132]
	MshA	*rv0486*	None	Adenosine derivatives and synthetic compounds	—	—	—	—	Research needs to be further	[133–136]
	MshC	*rv2130c*				—				
	PptT	*rv2794c*	None	8918	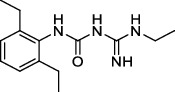	Inhibit PptT to block the activation of Pks13 enzyme and various type-І Pks	—	—	Short half-life	[137, 138]
	MptpA	*rv2234*	None	Natural products and synthetic compounds	—	—	—	—	The best inhibition activity at nM level	[139–142, 177]
	MptpB	*rv0153c*								
	Biofilm	*—*	None	C10	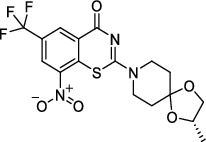	Prevent the formation of biofilm to make *Mtb* more susceptible to antibiotics	—	—	Research needs to be further	[145]

## Data Availability

The data used to support the findings of this study are included in this article.

## Abbreviations

8918: Amidino-urea 1-[(2,6-diethylphenyl)-3-N-ethylcarbamimodoyl]urea
Alr: Alanine racemase
DprE: Decaprenylphosphoryl-*β*-D-ribose-2′-epimerase
DdlA: D-Ala-D-Ala ligase
Ddn: Deazaflavin-dependent nitroreductase
FabH: *β*-Ketoacyl-acyl carrier protein synthase III
Ftsz: Filamenting temperature-sensitive protein Z
InhA: Enoyl-acyl carrier protein reductase
Ino1: Inositol-1-phosphate synthase
ICL: Isocitrate lyase
LCB01-0371: Delpazolid
LeuRS: Leucyl-tRNA synthase
MDR: Multidrug-resistant
*Mtb*: *Mycobacterium tuberculosis*
MurX: Translocase І
MRX-І: Contezolid
MenB: 1,4-Dihydroxy-2-naphthoyl CoA synthase
SK: Shikimate kinase
MmpL: Mycobacterial membrane protein large
MSH: Mycothiol
MIC: Minimum inhibitory concentration
NO: Nitric oxide
NDH-2: Type-II NADH dehydrogenase
NATs: Arylamine N-acetyltransferase
PA-824: Pretomanid
PUN-100480: Sutezolid
PptT: 4′-Phosphopantetheinyl transferase
STPK: Ser/Thr protein kinase
TB: Tuberculosis
WecA: GlcNAc-1-P transferase
WHO: Word Health Organization
XDR: Extensively drug-resistant.
